# Effect of Guibi-Tang, a Traditional Herbal Formula, on Retinal Neovascularization in a Mouse Model of Proliferative Retinopathy

**DOI:** 10.3390/ijms161226211

**Published:** 2015-12-16

**Authors:** Yun Mi Lee, Yu-Ri Lee, Chan-Sik Kim, Kyuhyung Jo, Eunjin Sohn, Jin Sook Kim, Junghyun Kim

**Affiliations:** KM Convergence Research Division, Korea Institute of Oriental Medicine, Daejeon 34054, Korea; candykong@kiom.re.kr (Y.M.L.); yrsanta@kiom.re.kr (Y.-R.L.); chskim@kiom.re.kr (C.-S.K.); jopd7414@kiom.re.kr (K.J.); ssen4022@kiom.re.kr (E.S.); jskim@kiom.re.kr (J.S.K.)

**Keywords:** retinal neovascularization, fibroblast growth factor 2, plasminogen activator inhibitor 1, vascular endothelial growth factor, oxygen-induced retinopathy

## Abstract

Ocular pathologic angiogenesis is an important causative risk factor of blindness in retinopathy of prematurity, proliferative diabetic retinopathy, and neovascular macular degeneration. Guibi-tang (GBT) is a frequently used oriental herbal formula in East Asian countries, and is also called Qui-pi-tang in Chinese and Kihi-To in Japanese. In the present study, we investigated the preventive effect of GBT on retinal pathogenic neovascularization in a mouse model of oxygen-induced retinopathy (OIR). C57BL/6 mice were exposed to 75% hyperoxia for five days on postnatal day 7 (P7). The mice were then exposed to room air from P12 to P17 to induce ischemic proliferative retinopathy. GBT (50 or 100 mg/kg/day) was intraperitoneally administered daily for five days (from P12 to P16). On P17, Retinal neovascularization was measured on P17, and the expression levels of 55 angiogenesis-related factors were analyzed using protein arrays. GBT significantly decreased retinal pathogenic angiogenesis in OIR mice, and protein arrays revealed that GBT decreased PAI-1 protein expression levels. Quantitative real-time PCR revealed that GBT reduced vascular endothelial growth factor (VEGF), fibroblast growth factor 2 (FGF2), and plasminogen activator inhibitor 1 (PAI-1) mRNA levels in OIR mice. GBT promotes potent inhibitory activity for retinal neovascularization by decreasing VEGF, FGF2, and PAI-1 levels.

## 1. Introduction

Retinal neovascularization, which is the pathological growth of new blood vessels, is associated with many disease processes including diabetic retinopathy, retinopathy of prematurity, central retinal vein occlusion, and branch retinal vein occlusion [1,2].

Vascular endothelial growth factor (VEGF) plays a central role in physiological and pathological angiogenesis [3]. However, despite strong evidence associating VEGF with retinal neovascularization, it is likely that VEGF collaborates with other angiogenic factors such as insulin-like growth factor-I (IGF-I) and fibroblast growth factor 2 (FGF2) to stimulate retinal neovascularization [4]. Experimental evidence indicates that targeting FGF2, much like VEGF, might result in a synergistic angiogenic response for the treatment of angiogenesis-related diseases (*in vitro* and *in vivo*) [5,6,7,8]. FGF2 has been a candidate retinal angiogenesis factor longer than VEGF, and many studies have investigated its possible role in retinal neovascularization [9]. Moreover, VEGF and FGF2 induced production of uPA and plasminogen activator inhibitor 1 (PAI-1) in cultured bovine endothelial cells [10,11]. PAI-1 belongs to the serine proteinase inhibitors (serpin) superfamily [12]; PAI-1 is known as an endogenous inhibitor of a major fibrinolytic factor and tissue-type plasminogen activator [13]. Inhibition or loss of PAI-1 downregulates overall retinal angiogenesis, which suggests that PAI-1 is a potential therapeutic target for retinal neovascularization [14].

The traditional herbal medicine Guibi-tang (Guipi-tang in Chinese or Kihi-to in Japanese), is a mixture of 12 herbs that are used to treat amnesia, fatigue, poor memory or forgetfulness, anorexia, anemia, insomnia, palpitation, and neurosis [15]. Recent evidence has suggested that Guibi-tang (GBT) has specific bioactivities, including immune regulation [16], anti-stress [17], antioxidant effects [18], and protective effect of the gastric mucosa [19]. Moreover, GBT is a Chinese patent formula for wet macular degeneration [20]. Decursin, a major ingredient in GBT, inhibited retinal neovascularization in a mouse model of retinopathy of prematurity [21]. Despite the various effects of GBT, knowledge about the mechanisms of its effect on retinal neovascularization is limited. To the best of our knowledge, there are no published studies describing the therapeutic effect of GBT on retinal neovascularization. Therefore, the aim of the current study is to examine the pharmacological effects of GBT on retinal angiogenesis in a mouse model of oxygen-induced retinopathy (OIR).

## 2. Results

### 2.1. GBT Treatment Significantly Downregulated the Central Non-Perfusion Area and Retinal Tufts in Flat Mounts

**Figure 1:**
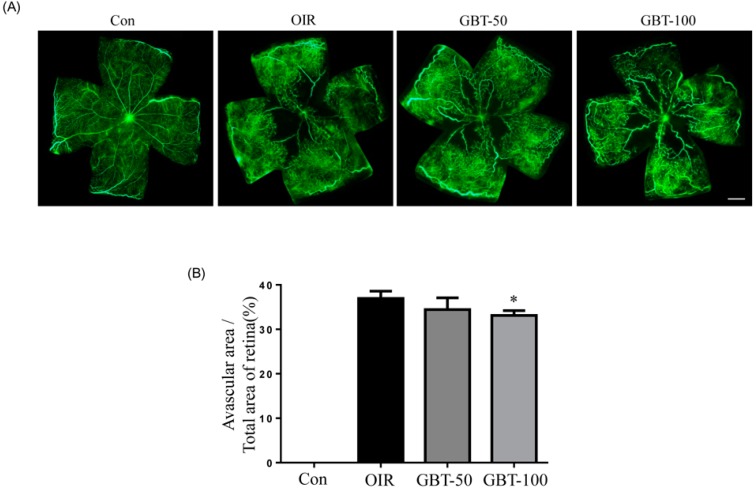
The effect of GBT on retinal neovascularization in OIR mice. (**A**) The retinal blood vessels were visualized via fluorescein angiography using FITC-dextran. Con, normal control mice; OIR, saline-treated OIR mice; GBT-50, OIR mice treated with 50 mg/kg of GBT; and GBT-100, OIR mice treated with 100 mg/kg GBT; Scale bar = 500 µm; (**B**) The quantification results are expressed as a percentage of the central nonperfused area within the total retinal area. The bar graph values represent the mean ± SE (*n* = 5). * *p* < 0.05 for OIR group *vs.* GBT-treated group.

Vascular development and neovascularization patterns were easily observed in the retinal flat-mounts that were prepared after fluorescein-dextran perfusion. The OIR mice that were treated with GBT exhibited significant decreases in ischemia retinopathy-induced pathological changes. Oxygen-induced retinal neovascularization was elicited by tissue ischemia because of retinal central capillary dropout during hyperoxia. As demonstrated in Figure 1, GBT promoted the revascularization of the central retina in OIR. Mice that were treated with 100 mg/kg GBT significantly changed the non-perfusion area in the retina center compared to the OIR group.

**Figure 2:**
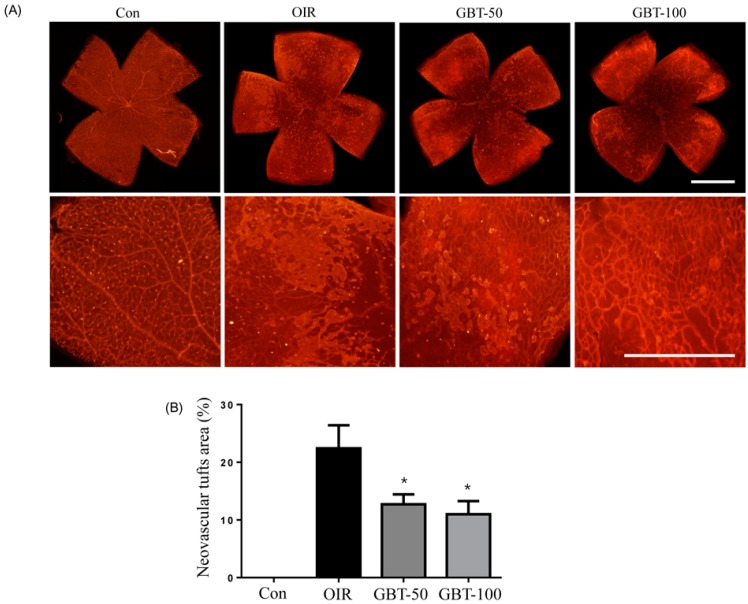
The effect of GBT on retinal neovascular tufts in OIR mice. (**A**) The retinal neovascular tufts were visualized using isolectin B4 staining. Con, normal control mice; OIR, saline-treated OIR mice; GBT-50, OIR mice treated with 50 mg/kg of GBT; and GBT-100, OIR mice treated with 100 mg/kg GBT; Scale bar = 500 µm; (**B**) Quantification results are expressed as neovascular tufts on the retina surface. The bar graph values represent the mean ± SE (*n* = 5). * *p* < 0.05 for OIR group *vs.* GBT-treated group.

Morphometric analysis of retinal flat mounts stained with TRITC–isolectin B4 was conducted to assess vessel growth (Figure 2). GBT treatment prevented pathogenic retinal neovascularization compared with the OIR group on P17. Both GBT doses significantly reduced neovascular tuft formation (by 43.3% and 51.0%, respectively) compared with the OIR group (Figure 2). Consequently, GBT treatment helped maintain a relatively normal retinal structure during ischemic retinopathy.

**Figure 3:**
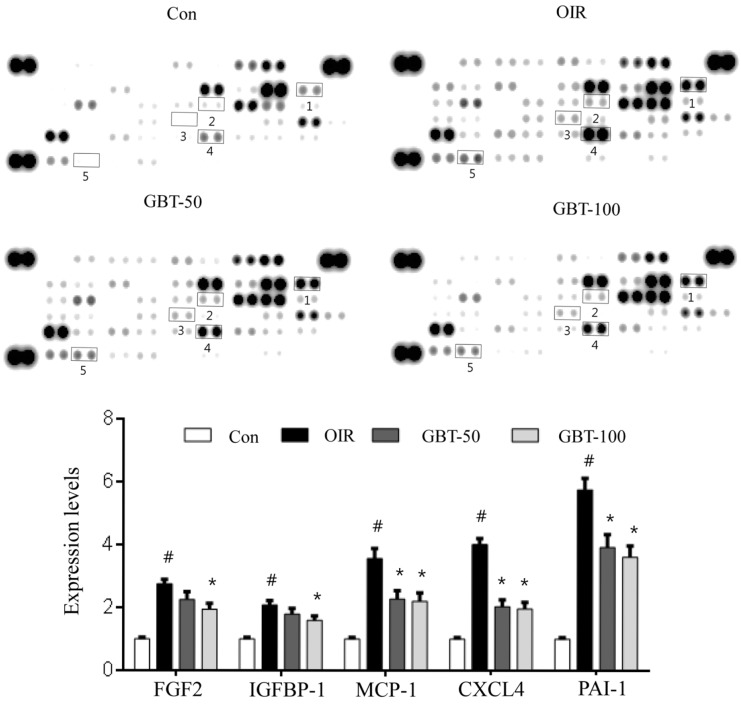
Expression of retinal angiogenesis-related proteins in OIR mice. Pro- and anti-angiogenic factor expression in the retina was analyzed using protein arrays and quantified using ImageJ software. The negative control is located in the lower right corner of the arrays, and the positive controls are located in other three corners. The proteins for which expression was modulated in GBT-treated retinas are indicated by numbers. Con, normal control mice; OIR, saline-treated OIR mice; GBT-50, OIR mice treated with 50 mg/kg GBT; and GBT-100, OIR mice treated with 100 mg/kg GBT. 1: FGF2, fibroblast growth factor 2; 2: IGFBP-1, insulin-like growth factor binding protein 1; 3: MCP-1, Monocyte Chemoattractant Protein-1; 4: CXCL4, chemokine (C-X-C motif) ligand 4; 5: PAI-1, Plasminogen activator inhibitor-1. Data are represented as mean ± SE (*n* = 4). ^#^ *p* < 0.0001 for control group *vs.* OIR group; * *p* < 0.05 for OIR group *vs.* GBT-treated group.

### 2.2. GBT Significantly Reduced Angiogenesis-Related Factor Protein Expressions

To identify mechanistic insights into the effects of GBT in OIR mice, we analyzed the expression levels of 55 angiogenesis-related proteins that play roles in OIR pathogenesis via the protein array of the total protein isolated from the retinas. As demonstrated in Figure 3, GBT treatment at a dose of 100 mg/kg significantly decreased the expression of pro-angiogenic factors (*i.e.*, MCP-1, IGFBP-1, PAI-1, and FGF2) compared with saline-treated OIR mice. Consistent with a previous study [22], VEGF protein was not detected in the angiogenesis protein array, possibly due to the low sensitivity to this antibody on the array. Thus, we further investigated changes in VEGF mRNA expression levels, which is a key player in angiogenesis.

### 2.3. GBT Treatment Downregulates VEGF, FGF2 and PAI-1 mRNAs Expression

**Figure 4:**
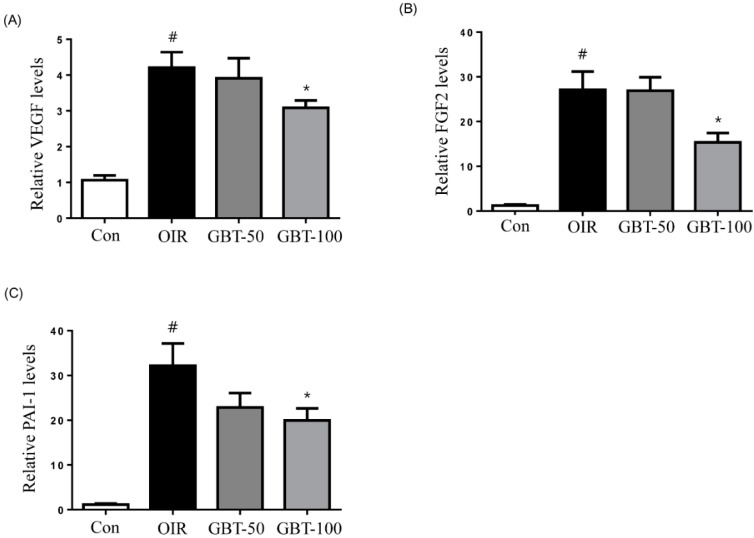
Real-time PCR analysis of VEGF, FGF2 and PAI-1 mRNA levels in OIR mice. When compared with normal controls, relative VEGF (**A**); FGF2 (**B**) and PAI-1 (**C**) mRNA expression levels were markedly increased in OIR mouse retinas and dramatically reduced after GBT treatment. Con, normal control mice; OIR, saline-treated OIR mice; GBT-50, OIR mice treated with GBT (50 mg/kg); and GBT-100, OIR mice treated with GBT (100 mg/kg). Data are represented as mean ± SE (*n* = 4). ^#^ *p* < 0.0001 for control group *vs.* OIR group; * *p* < 0.05 for OIR group *vs.* GBT-treated group.

Retinal VEGF, FGF2, and PAI-1 mRNA expressions were examined using real-time PCR. As expected, we observed a robust induction of VEGF mRNA during oxygen-induced retinopathy. In addition, FGF2 and PAI-1 mRNA levels were elevated in OIR mice compared with normal control mice. However, VEGF, FGF2 and PAI-1 mRNA levels were significantly decreased in 100 mg/kg GBT-treated OIR mice (Figure 4).

## 3. Discussion

Retinal neovascularization is a major cause of blindness worldwide. Laser photocoagulation, cryotherapy, and intravitreal injection of anti-VEGF antibodies and VEGFR inhibitors are well-established treatments for retinal neovascularization. However, these retinal neovascularization therapies are limited and involve invasive procedures. Thus, better retinal neovascularization treatments are still very much in demand. In the present study, we evaluated the anti-angiogenic effect of GBT during pathological retinal neovascularization in OIR mice. We demonstrated that GBT has anti-angiogenic effects in this animal model.

The OIR model has been widely used as a valuable tool in retinal neovascularization pathogenesis research [23]. In the mouse OIR model, the hyperoxic phase (P7–P12) results a loss of immature vessels in the central retina and development of vaso-obliteration (VO). When the mice are returned to room air (P12–P17), the retina becomes hypoxic due to the absence of the retinal vasculature, which stimulates pro-angiogenic factors and results in abnormal neovascularization [24]. Many previous studies using the OIR model have demonstrated valuable effects and laid the foundation for today’s clinical application of anti-angiogenic treatments in retinal neovascularization [23,24].

Clinical evidence has repeatedly suggested the use of anti-VEGF or VEGFR inhibitors. Bevacizumab, a humanized monoclonal antibody targeting VEGF and the tyrosine kinase inhibitors sorafenib, a multikinase inhibitor targeting VEGFR/platelet-derived growth factor receptor (PDGFR) represent the first approved anti-angiogenic agents by the FDA that suppress neovessel formation [25]. However, despite anti-VEGF treatment, components of FGF, PDGF, epidermal growth factor (EGF), and other pathways may compensate for a VEGF blockade and promote angiogenesis [26]. Previous studies have reported that tumors may acquire resistance to anti-VEGF therapy following angiogenesis reactivation, which is induced by compensatory upregulation of FGF2/FGFR system in experimental animals [27] and patients with cancer [28]. Thus, accumulated reports have suggested that FGF has a significant role in retinal pathogenic angiogenesis. Accordingly, there is a strong need to develop new, affordable drugs with different modes of action [29]. A combination of multiple herbs has a synergistic effect due to the phytocompounds contained in the different herbs [30]. Interestingly, GBT, which is a mixture of 12 herbs, inhibits retinal neovascularization via the suppression of VEGF, FGF2, and PAI-1 in OIR mice. Our array data demonstrate that GBT exerts an inhibitory effect on angiogenesis by downregulating the expression of FGF2, IGFBP-1, MCP-1 and PAI-1. Although CXLCL4, an anti-angiogenic factor, was also significantly downregulated by GBT treatment, the upregulation of CXCL4 in saline-treated OIR mice may be a defense mechanism against angiogenesis. A decrease of CXCL4 in GBT-treated OIR mice might be an indirect effect as a result of reduced angiogenesis.

FGF2 is a member of heparin-binding growth factors and a potent pro-angiogenic signaling molecule *in vivo* and *in vitro*. FGF2 is expressed in many tissues and cell types and directly affect new vessel growth through heparan-sulfate proteoglycan tyrosine kinase receptors and integrins that are expressed on the surface of vascular endothelial cells [31,32]. Furthermore, blocking both FGF2 and VEGF is essential to disrupting the interconnection between these two angiogenic factors and effectively inhibiting early stage angiogenesis [33]. Recently, novel anti-angiogenic drugs have been developed that block the mechanisms of FGF2 and VEGF action [34]. Previous studies have demonstrated that anti-FGF2 antibodies either alone or in combination with anti-VEGF antibodies strongly upregulate PAI-1 expression in bovine aortic endothelial cells and bovine vascular endothelial cells and [10,35]. PAl-1 production plays a role in mediating the trophic effects resulting from FGF2 receptor activation by FGF2 itself or through the action of the cell adhesion molecule. PAI-1 can both inhibit and promote angiogenesis [13]. PAI-1 inhibited endothelial sprouting in an *ex vivo* study [36]. In contrast, PAI-1 promotes tumor growth via anti-apoptotic effects in cancer cells as well as non-cancer cell lines *in vitro* [37]. It was also demonstrated that a lack of host PAI-1 suppresses tumor growth [38] and inhibits cancer metastasis and angiogenesis [39]. In OIR mice, PAI-1 protein and mRNA expression levels are significantly elevated during active phases of new vessel growth [14]. Therefore, VEGF, FGF2, and PAI-1 have been proposed as targets for treating retinal neovascularization. GBT could reduce VEGF, FGF2, and PAI-1 expression in OIR mice. These observations indicate that the inhibitory effect of GBT on retinal neovascularization might occur via the partial inhibition of VEGF, FGF2, and PAI-1. 

VEGF inhibitors are of great benefit to patients with neovascular ocular diseases [40]. However, an increasing amount of evidence suggests that IGFBP-1 and MCP-1 also have a role in retinal neovascularization. Our array results indicate that GBT inhibits the expression of IGFBP-1 and MCP-1. MCP-1 expression is highly increased in diabetic retinopathy, while MCP-1 deficiency prevents the development of subretinal neovascularization in MCP^−/−^ mice [41]. The enhanced expression of IGFBP-1 in the retina also plays an important role in the pathogenesis of retinal neovascularization. Vitreal expression levels of IGFBP-1 were increased in patients with ischemic central retinal vein occlusion [42], and IGFBP-1 shows large increases in neovascular tufts in ischemic retinopathy [43]. Based on these findings, we can hypothesize that IGFBP-1 and MCP-1 may be valid secondary targets when fighting retinal neovascularization [40]. In the present study, GBT prevented retinal neovascularization through the downregulation of IGFBP-1 and MCP-1. 

In OIR animal models, reactive oxygen species (ROS) generation during ischemia plays a crucial role in retinal neovascularization. Ischemic retina leads to excessive production of ROS, which, in turn, activates NADPH oxidase and contributes to retinal pathogenic neovascularization [44]. Natoli *et al.* reported that oxidative stress-related retinal cell death and photoreceptor dysfunctions were also observed in OIR animal models [45]. Excess ROS disturbs the normal mitochondrial function by widening the permeability transition pore located on an inner mitochondrial membrane, resulting in further ROS overproduction [46], the release of cytochrome c, and the depletion of ATP, leading to cell damage [47]. Cellular ATP is mainly generated in mitochondria. Mitochondrial F_0_F_1_-ATPase synthesizes ATP during oxidative phosphorylation, which is important for cellular survival and proliferation [48]. Recently, Yamamoto *et al.* reported that several subunits of the mitochondrial F_0_F_1_-ATPase are expressed on the surface of the human umbilical vein endothelial cells (HUVEC) [49]. Moser *et al.* showed that angiostatin, a potent anti-angiogenic agent, suppressed the proliferation if HUVECs by reducing ATPase activity [50]. More recently, Calzia *et al.* reported that polyphenolic phytochemicals such as resveratrol and curcumin inhibited the mitochondrial F_0_F_1_-ATP synthase in the rod outer segments of the retina [51]. GBT has various polyphenolic compounds such as liquiritin and glycyrrhizin. However, the effect of GBT on F_0_F_1_-ATP synthase has not yet been investigated.

GBT contains 12 herbal medicines, and its ingredient compounds are two flavonoids (liquiritin and glycyrrhizin), a phenolic (6-gingerol) and five coumarins (nodakenin, nodakenetin, decursinol, decursinol angelate, and decursin) [52,53]. Glycyrrhizin has anti-angiogenic activities such as endothelial cell migration, invasion and tube formation, along with anti-tumor activities such as angiogenesis and tumor growth in mice [54]. Moreover, glycyrrhizin, which is a selective inhibitor of high mobility group box-1, reduced retinal neovascularization in OIR mice [55]. The major coumarins decursin, decursinol and decursinol angelate exert inhibitory effects on angiogenesis by reducing VEGF expression [56,57]. Cloricromene, a semi-synthetic coumarin derivative, lowered retinal expressions of VEGF, ICAM-1, and nitrotyrosine in STZ-induced diabetic rats [58]. Eriodictyol, a flavonoid compound, protected human retinal pigment epithelial cells from oxidative stress-induced cell death [59] and reduced retinal expressions of TNF-α, ICAM-1, VEGF, and eNOS in STZ-induced diabetic rats [60]. Flavonoids inhibited VEGF/FGF2-induced angiogenesis to prevent VEGF/FGF2-induced uPA expression and the activation of its inhibitor PAI-1 [61].

Herbal medicines usually consist of many compounds. Generally, herbal practitioners rarely use single herb alone. Herbal formula has many advantages, such as additive synergic interactions among a variety of phytocompounds contained in the different herbs [62]. Although it was still unclear which compound in GBT might play the most important role in the inhibition of retinal neovascularization, these observations suggest that the prevention of retinal neovascularization by GBT may occur due to a combination of these compounds’ effects. However, the detailed mechanism that underlies the synergistic effects of GBT on retinal neovascularization remains unknown.

## 4. Experimental Section

### 4.1. GBT Preparation

GBT consists of 12 herbal medicines, which were purchased from Baekjedang (Daejeon, Korea). The mixture of crude herbs was boiled with distilled water at 100 °C for 2 h in the following ratio: *Angelica gigas* (3.75 g), *Dimocarpus longan* (3.75 g), *Panax ginseng* (3.75 g), *Zizyphus jujuba* (3.75 g), *Polygala tenuifolia* (3.75 g), *Astragalus membranaceus* (3.75 g), *Poria cocos* (3.75 g), *Atractylodes japonica* (3.75 g), *Zizyphus jujube* (3.75 g), *Aucklandia lappa* (1.875 g), *Glycyrrhiza uralensis* (1.125 g), and *Zingiber officinale* (6.25 g). The extract was filtered, lyophilized and stored at −20 °C until use. A standardized powder of GBT was provided by Hyeun-Kyoo Shin (Korea Institute of Oriental Medicine, Daejeon, Korea). The HPLC fingerprint and contents of three major compounds of GBT are described in a previous report [52]. The quantity of the three compounds in GBT were 0.70 ± 0.01 mg/g (liquiritin), 0.03 ± 0.01 mg/g (glycyrrhizin), and 1.43 ± 0.01 mg/g (nodakenin).

### 4.2. A Mouse Model of Oxygen-Induced Retinopathy

Ischemic retinopathy was induced in C57BL/6 mouse pups, as described previously [55]. On postnatal day 12 (P12), after being exposed to 75% ± 2% oxygen for five days (P7–P12), the mice were randomly assigned to one of three groups: OIR, GBT-50 (50 mg/kg/day), or GBT-100 (100 mg/kg/day). The normal control group (Con) was simultaneously raised in normal room air. The GBT was dissolved in saline, and 100 µL of this solution was injected intraperitoneally once daily for five days (P12–P16). The OIR and normal control groups were injected with saline solution for five days. On P17, after five days of intraperitoneal injections, the mice were anesthetized and sacrificed. These experiments were repeated four times using four animals per group. All animal experiments were approved by the Korea Institute of Oriental Medicine Institutional Animal Care and Use Committee. (Approval number: 14-024, approval date: 20 February 2014).

### 4.3. Fluorescein-Dextran Microscopy

On P17, the mice were deeply anesthetized using zolazepam (Zoletil, Virbac, Carros, France), and PBS containing fluorescein-dextran (FD40S, Sigma-Aldrich, St. Louis, MO, USA) was subsequently perfused via the left ventricle. The retinas were dissected, flat mounted on to glass slides and viewed using fluorescence microscopy (BX51, Olympus, Tokyo, Japan). The area of non-perfusion in the retinal center was quantified using ImageJ software (NIH, Bethesda, MD, USA).

### 4.4. Lectin Staining

The retinas were incubated with 1% bovine serum albumin and 5% Triton X-100 in PBS for three hours at room temperature. The retinas were washed three times with PBS and incubated overnight at 4 °C with *Bandeiraea simplicifolia* isolectin B4 (Sigma-Aldrich, St. Louis, MO, USA), which was diluted 1:50 in PBS. The retinas were washed with 0.05% Tween 20 in PBS, followed by incubation with streptavidin TRITC (1:500, Serotec, Oxford, UK) for 4 h at 37 °C. The retinas were flat mounted and viewed using fluorescence microscopy (BX51, Olympus, Tokyo, Japan). ImageJ software (NIH, Bethesda, MD, USA) was used to measure the neovascular tufts on the retinas.

### 4.5. Angiogenesis-Related Protein Array

To investigate angiogenesis-related proteins in the retinas, a mouse angiogenesis array (R&D Systems Inc., Minneapolis, MN, USA) was performed according to the manufacturer’s protocol. Briefly, pooled mouse retinas (*n* = 3) were homogenized in PBS using protease inhibitors, centrifuged at 10,000× *g* for 5 min, and total protein concentrations were quantified. Lysates were added to a membrane and blotted with antibodies against angiogenesis-related proteins. After incubation overnight at 4 °C, the membranes were treated with streptavidin-horseradish peroxidase and visualized using an enhanced chemiluminescence detection system (Amersham Bioscience, Piscataway, NJ, USA) on an image analyzer (LAS-3000, Fujifilm, Tokyo, Japan). Optical density measurements were obtained using ImageJ software (NIH, Bethesda, MD, USA).

### 4.6. Real-Time PCR Analysis

Total RNA was isolated using the TRIzol reagent (Invitrogen, Carlsbad, CA, USA), and 0.5 μg of total RNA was reverse transcribed into cDNA with the PrimeScript First Strand cDNA Synthesis kit (Bio-Rad, Hercules, CA, USA). Quantitative real-time PCR was performed with specific primers for VEGF, FGF2, PAI-1 and GAPDH using an iQ5 Continuous Fluorescence Detector System (Bio-Rad, Hercules, CA, USA). The primer sequences were as follows: VEGF, 5′-TCCTCCTATCTCCACCACCTATCC-3′ and 5′-GACCCAGCCAGCCATACCC-3′; FGF2, 5′-AGAGCGACCCACACGTCAAAC-3′ and 5′-CCAACTGGAGTATTTCCGTGACC-3′; PAI-1, 5′-GCCAGATTTATCATCAATGACTGGG-3′ and 5′-GGAGAGGTGCACATCTTTCTCAAAG-3′; GAPDH, 5′-TGTCGTCCCAGTTGGTAA-3′ and 5′-CTTTGCAGCTCCTTCGTT-3′. All real-time PCR experiments were run as triplicates. GAPDH mRNA levels were determined for the normalization of VEGF, FGF2 and PAI-1 mRNA expression values using the iQ5 optical system software (Bio-Rad, Hercules, CA, USA).

### 4.7. Statistical Analysis

The results were expressed as the mean ± SE and analyzed using one-way analysis of variance (ANOVA) followed by either Tukey’s multiple comparison test or an unpaired Student’s *t*-test. All analyses were performed using Prism 6.0 software (GraphPad Software, San Diego, CA, USA).

## 5. Conclusions

In conclusion, we have demonstrated that GBT inhibited ischemic retinopathy-induced retinal pathogenic angiogenesis in OIR mice for the first time. In addition, VEGF, FGF2 and PAI-1 overexpression were significantly reduced by GBT treatment. These observations suggest that GBT has anti-angiogenic activities through the partial inhibition of VEGF, FGF2, and PAI-1. Further studies must be carried out to figure out the potential clinical use of GBT.
